# Modeling and Experimental Analysis on the Temperature Response of AlN-Film Based SAWRs

**DOI:** 10.3390/s16081205

**Published:** 2016-07-30

**Authors:** Shuo Chen, Zheng You

**Affiliations:** 1 Department of Precision Instrument, Tsinghua University, Beijing 100084, China; chenshuo_tsinghua@126.com; 2 State Key Laboratory of Precision Measurement Technology and Instruments, Tsinghua University, Beijing 100084, China

**Keywords:** surface acoustic resonators, aluminum nitride, equivalent circuit modeling, high temperature response

## Abstract

The temperature responses of aluminum nitride (AlN) based surface acoustic wave resonator (SAWR) are modeled and tested. The modeling of the electrical performance is based on a modified equivalent circuit model introduced in this work. For SAWR consisting of piezoelectric film and semiconducting substrate, parasitic parameters from the substrate is taken into consideration for the modeling. By utilizing the modified model, the high temperature electrical performance of the AlN/Si and AlN/6H-SiC based SAWRs can be predicted, indicating that a substrate with a wider band gap will lead to a more stable high temperature behavior, which is further confirmed experimentally by high temperature testing from 300 K to 725 K with SAWRs having a wavelength of 12 μm. Temperature responses of SAWR’s center frequency are also calculated and tested, with experimental temperature coefficient factors (TCF) of center frequency being −29 ppm/K and −26 ppm/K for the AlN/Si and AlN/6H-SiC based SAWRs, which are close to the predicted values.

## 1. Introduction

Surface acoustic wave resonators (SAWR) are widely used in sensing and telecommunicating industries [1,2,3,4]. Most of the conventional SAWR devices are based on bulk piezoelectric materials such as quartz, LiNbO_3_, and LiTaO_3_, but the decomposition or the weakening of piezoelectricity limits their working temperature within a relatively low range [5,6], thus novel piezoelectric materials with good thermal stabilities are valuable for high temperature applications. Several literatures have summarized that novel piezoelectric materials such as langasite, GaPO_4_, and YCa_4_O(BO_3_)_3_·(or YCOB) [5,7] are promising for high temperature environment. However, the majority of them are bulk materials which are less conventional and more expensive than film-like materials.

Aluminum nitride (AlN), is a non-ferroelectric piezoelectric material with a high melting point, high thermal conductivity [7,8], being compatible with most commonly seen CMOS fabrication processes [9,10,11]. Besides, AlN can be deposited on commercialized substrates such as Silicon (Si), hexagonal Silicon Carbide (α-SiC), poly-crystalline cubic Silicon Carbide, diamond and sapphire [12,13,14,15,16]. Among these aforementioned substrates, Si and α-SiC are probably the most compatible ones with conventional micro-fabrication processes. Therefore it is of interest to study the temperature responses of SAWRs based on AlN/Si and AlN/α-SiC in respect of the electrical property and center frequency.

**Figure 1:**
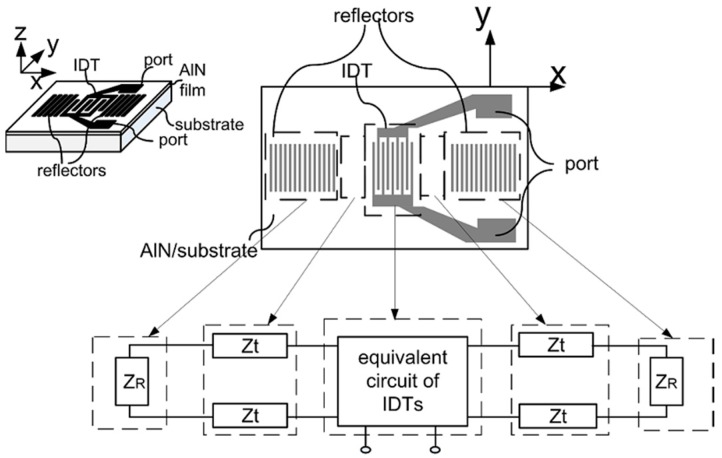
The equivalent circuit model of SAWR.

In previous literatures, the equivalent circuit model of SAWR (Figure 1) has been developed and used to predict the electrical performance of SAWR [17,18,19]. As can be seen, the equivalent circuit of SAWR consists of the equivalent circuit components representing the inter-digital transducer (IDT) part, the reflectors on both sides (Z_R_), the transmission lines between IDT and reflectors (Z_t_), and the port connected with IDT, among which, the equivalent circuit of the IDT part is established to describe the electro-mechanical coupling between the alternating current (AC) signal and SAW.

For the AlN/Si and AlN/α-SiC based SAWRs in this work, the IDT parts of the devices which are connected with the electrical port will be affected by the parasitic parameters from the semiconducting substrate (Si, 6H-SiC as one of α-SiC’s poly-types in this work), hence the aforementioned classic equivalent circuit shall be modified to take this effect into consideration. This work introduces the modification of the equivalent circuit model by analyzing the origins of the parasitic parameters and their equivalent electrical components. The modified equivalent circuit model is further used for calculating the electrical performance of the AlN film based SAWRs.

The temperature coefficient factor (TCF) of SAWR’s center frequency is another important aspect of temperature responses. In this work, the TCF of each composing material (AlN, Si, and 6H-SiC) is calculated for a prediction of SAWR’s TCF, and the influence from the thermal mismatch between AlN and the substrate is analyzed.

## 2. Modeling

### 2.1. Modification of the Equivalent Circuit of IDT

**Figure 2:**
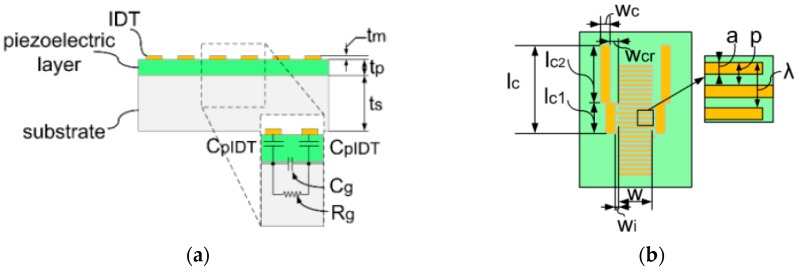

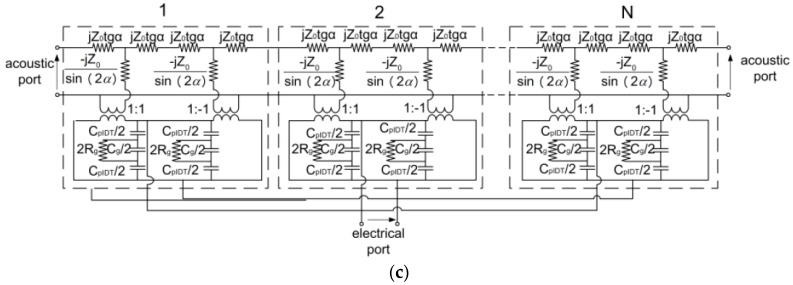
Modified equivalent circuit model of the IDT part. (**a**) Schematic view of the SAWR and the equivalent circuit components; (**b**) Layout and geometric definitions of SAWR in this work; (**c**) Modified equivalent circuit of N-pair IDT.

With the existence of the aforementioned parasitic parameters from the semiconducting substrate, the equivalent electrical components between IDT grids are modified, as shown in Figure 2a, where C_pIDT_ represents the capacitor of the piezoelectric layer under the IDT electrodes, R_g_ represents the resistor from the substrate at the gap between the IDT electrodes, and C_g_ represents the capacitor from the substrate and piezoelectric layer at the same area.

Thus the equivalent circuit model of the IDT part can be derived by combining the equivalent circuits of single pair IDT, as shown in Figure 2c. The acoustic ports of single IDT are connected in serial and the electrical ports are connected in parallel. With the geometric definitions of the SAWR in Figure 2b, the equivalent circuit components can be given as:

(1) $$R_{g}=\frac{R_{□}(p-a)}{w}$$

(2) $$C_{g}=\frac{\varepsilon_{0}\varepsilon_{p}{\mathrm{wt}}_{p}}{p-a}+\frac{\varepsilon_{0}\varepsilon_{s,c}{\mathrm{wt}}_{s}}{p-a}$$

(3) $$C_{\mathrm{pIDT}}=\frac{\varepsilon_{0}\varepsilon_{p}\mathrm{aw}}{t_{p}}$$

where R_□_ is the square resistance of the substrate, t_p_ is the thickness of the piezoelectric layer, t_s_ is the thickness of the substrate, ε_s,c_ and ε_p_ are the complex relative permittivity of the substrate and the piezoelectric layer, respectively. Z_0_ is the equivalent electrical impedance representing the acoustic impedance from the piezoelectric layer and the substrate, and has been given by [20] as:

(4) $$Z_{0}=\frac{2\pi }{\omega_{0}C_{s}K^{2}}$$

where ω_0_ is the angular resonant frequency of SAWR, C_s_ is the static capacitor storing the electrical energy between the IDT electrodes, and can be given by Equation (5), where C_g,r_ is the real part of C_g_:

(5) $$C_{s}=(2{C_{\mathrm{pIDT}}}^{-1}+{C_{g,r}}^{-1}{)}^{-1}$$

K^2^ is the electrical-mechanical coupling coefficient of the piezoelectric material, and can be derived experimentally through the serial and parallel resonant frequencies (f_s_ and f_p_, respectively) of the SAWR [21,22]:

(6) $$K^{2}=(\pi f_{s}/2f_{p})\cot(\pi f_{s}/2f_{p})$$

### 2.2. Parallel Parasitic Parameters Introduced from the Conducting Lines

**Figure 3:**
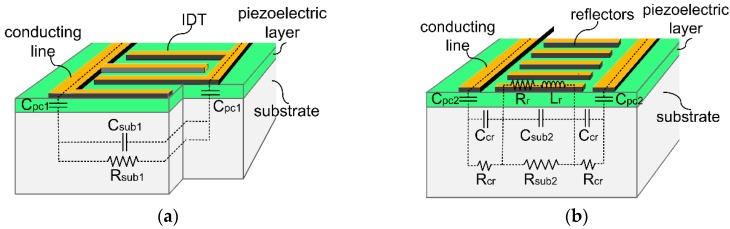
Parallel parasitic parameters from the piezoelectric film and the semiconducting substrate (**a**) IDT part; (**b**) Reflector part.

The semiconducting substrate will also introduce parasitic parameters from the conducting lines which connect the IDT and the port of the stimulating signal. The equivalent circuit components of these parasitic parameters are shown in Figure 3.

As can be seen from Figure 3, both the IDT part (Figure 3a) and reflector part (Figure 3b) will introduce parasitic effects from the substrate.

In the IDT part, each components are outlined as follows: C_pc1_ represents the capacitor from the piezoelectric film under the conducting lines, C_sub1_ represents the capacitor from the substrate between the conducting lines, and R_sub1_ represents the resistor from the substrate at the same region.

In the reflector part, C_pc2_ represents the same as defined in the IDT part; R_r_ represents the resistor of the reflecting grids; R_sub2_ and R_cr_ represent the resistors from the substrate lying under and outside the reflector region, respectively; C_sub2_ and C_cr_ represent the capacitor from the substrate at the same aforementioned area, respectively; L_r_ and R_r_ represents the inductance and resistance from the reflecting grids, respectively.

For these aforementioned parallel parasitic parameters, the analytical formulas can be approximately given as below, among which, L_r_ is calculated based on the inductance of a flat conductor [23,24]:

(7) $$C_{\mathrm{pc}1}=\frac{\varepsilon_{0}\varepsilon_{p}l_{c1}w_{c}}{t_{p}}$$

(8) $$C_{\mathrm{sub}1}=\frac{\varepsilon_{0}\varepsilon_{s,c}l_{c1}t_{s}}{2\left(w+2w_{i}\right)}+\frac{\varepsilon_{0}\varepsilon_{s,c}l_{c1}t_{s}}{2w_{i}}$$

(9) $$R_{\mathrm{sub}1}={\left({\left(\frac{R_{□}\left(w+2w_{i}\right)}{l_{c1}/2}\right)}^{-1}+{\left(\frac{R_{□}w_{i}}{l_{c1}/2}\right)}^{-1}\right)}^{-1}$$

(10) $$C_{\mathrm{pc}2}=\frac{\varepsilon_{0}\varepsilon_{p}l_{c2}w_{c}}{t_{p}}$$

(11) $$R_{\mathrm{sub}2\;}=\frac{R_{□}w}{l_{c2}}$$

(12) $$R_{\mathrm{cr}}=\frac{R_{□}w_{\mathrm{cr}}}{l_{c2}}$$

(13) $$C_{\mathrm{sub}2}=\frac{\varepsilon_{0}\varepsilon_{s,c}l_{c2}t_{s}}{w}$$

(14) $$C_{\mathrm{cr}}=\frac{\varepsilon_{0}\varepsilon_{s,c}l_{c2}t_{s}}{w_{\mathrm{cr}}}$$

(15) $$R_{r}=\frac{\rho_{m}w}{t_{m}{\mathrm{aN}}_{r}}$$

(16) $$L_{r}=\frac{\mu_{0}}{2\mathrm{Nr}\pi }w[\ln\left(\frac{2w}{{a+t}_{m}}\right)\;+0.5+0.2235(\frac{{a+t}_{m}}{w})]$$

They can be equivalently expressed as an impedance, namely Z_ep_, which is connected in parallel with the SAWR. Z_ep_ can be expressed as:

(17) $$Z_{\mathrm{ep}}=R_{\mathrm{ep}}+{\mathrm{jX}}_{\mathrm{ep}}$$

### 2.3. Modified Equivalent Circuit of AlN/Si SAWR

**Figure 4:**
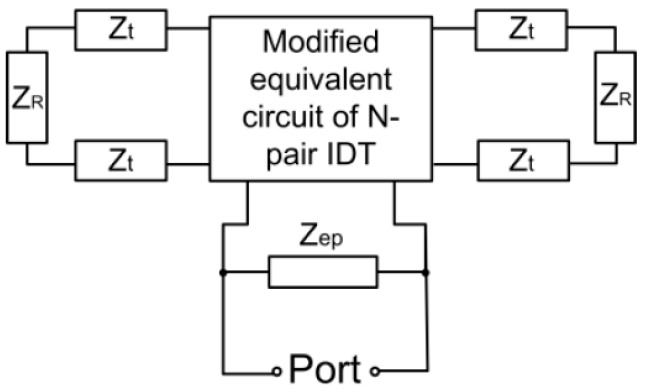
Modified equivalent circuit model.

The equivalent circuit model can thus be modified as shown in Figure 4, where the modified equivalent circuit has been shown in Figure 2c. Based on the modified model, the performance of AlN/Si based SAWR can be predicted and compared with the experimental results.

### 2.4. Temperature Response of Electrical Performance Based on the Modified Equivalent Circuit Model

Temperature will affect the performance of SAWR devices in a complex way due to its coupling with various material properties. These temperature-coupled material properties can be categorized into functioning parameters (such as the electrical-mechanical coupling coefficients), electrical parameters (such the resistivity, relative permittivity) and mechanical parameters (such as elastic constants, mass density). Here in this section, the temperature influence on the SAWR from the aspect of substrate will be analyzed based on the modified equivalent circuit model introduced above.

As can be seen from Section 2.1 and Section 2.2, the parasitic parameters introduced by the semiconducting substrate are related to the square resistance R_□_, the complex relative permittivity ε_s,c_, and the geometric parameters, among which, R_□_ can further be calculated through the resistivity ($\rho_{s}$) and the thickness of the substrate ($t_{s}$) as:

(18) $$R_{□}=\frac{\rho_{s}}{t_{s}}$$

Therefore the parasitic parameters influenced by the temperature can further be expressed as functions of $\rho_{s,T}$, ε_s,c,T_, and a_T_, being the temperature-dependent resistivity of substrate, temperature-dependent complex relative permittivity of substrate, thermal expansion coefficient, respectively. a_T_ is introduced here due to the variation of geometric parameters from thermal expansion.

For n-type semiconductors discussed here, $\rho_{s,T}$ can further be expressed as [25]:

(19) $$\rho_{s,T}={\left({e\mu }_{n,T}n_{T}\right)}^{-1}$$

where $\mu_{n,T}$ and $n_{T}$ are, respectively, the carrier mobility and electron concentration, both being temperature dependent, while e is the elementary charge and not sensitive to temperature. For a dopant concentration $N_{d}$ lower than $10^{14}$, $\mu_{n,T}$ can further be expressed as [25]:

(20) $$\mu_{n,T}\;=\mu_{300}{\left(\frac{T}{300}\right)}^{-2.2}$$

where $\mu_{300}$ is the carrier mobility of the substrate under 300 K, and T is the absolute temperature in K. The temperature dependent electron concentration $n_{T}$ can be expressed as [25]:

(21) $$n_{T}=\frac{N_{d}}{2}+\sqrt{\frac{{N_{d}}^{2}}{4}+{n_{i,T}}^{2}}$$

where $n_{i,T}$ is the intrinsic concentration of electrons, and can further be expressed as [25]:

(22) $$n_{i}\left(T\right)=\sqrt{N_{c,T}N_{v,T}}e^{-\frac{E_{g}}{2\mathrm{kT}}}$$

where $E_{g}$ is the band gap of the substrate, and k is the Boltzmann constant. $N_{c,T}$ and $N_{v,T}$ are, respectively, the effective density of states for the conduction band and valence band, and can be expressed as [25]:

(23) $$N_{C,T}=N_{C0}T^{3/2}$$

(24) $$N_{V,T}=N_{V0}T^{3/2}$$

where $N_{C0}$ and $N_{V0}$ are the effective density of states coefficients for the conduction band and valence band. The temperature dependent resistivity $\rho_{s,T}$ can thus be given as Equation (25) by combining Equations (19)–(24).

(25) $$\rho_{s,T}={\left({e\mu }_{300}{\left(\frac{T}{300}\right)}^{-2.2}\left(\frac{N_{d}}{2}+\sqrt{\frac{{N_{d}}^{2}}{4}+N_{C0}N_{V0}T^{3}e^{-\frac{E_{g}}{\mathrm{kT}}}}\right)\right)}^{-1}$$

The complex relative permittivity ε_s,c,T_ can be expressed by its real part $\varepsilon_{s,r}$ and imaginary part $\varepsilon_{s,i,T}$ as [26]:

(26) $$\varepsilon_{s,c,T}=\varepsilon_{s,r}-{j\varepsilon }_{s,i,T}$$

while the real part of $\varepsilon_{s,c,T}$ is the dielectric constant (ε_s,R_) of the substrate, the imaginary part, being temperature dependent, can be expressed as Equation (27) without considering the electronic and ionic polarization [26], where $\varepsilon_{0}$ is the dielectric constant of vacuum.

(27) $$\varepsilon_{s,i,T}=\frac{1}{2{\pi \varepsilon }_{0}{f\rho }_{s,T}}$$

As can be seen from Equation (27), $\varepsilon_{s,i,T}$ is a function of $\rho_{s,T}$ and frequency f, where $\rho_{s,T}$ has been given by Equation (25).

Based on the expressions listed above, the temperature response of substrate’s properties can be calculated. The chosen materials of the substrates are Si and 6H-SiC, with the nominal resistivity at 300 K ($\rho_{s,300}$) varying from 10 Ωcm to 10^4^ Ωcm. The material parameters used in calculation are listed in Table 1.

The intrinsic concentrations of electrons at 300 K ($n_{i,300}$) are firstly calculated by Equation (22), and the dopant concentrations ($N_{d}$) can further be calculated by combing Equations (19)–(21) as:

(28) $$\frac{1}{\rho_{s,300}}={e\mu }_{300}\left(\frac{N_{d}}{2}+\sqrt{\frac{{N_{d}}^{2}}{4}+{n_{i,300}}^{2}}\right)$$

The calculated $N_{d}$ with different $\rho_{s,300}$ are listed in Table 2.

**Figure 5:**
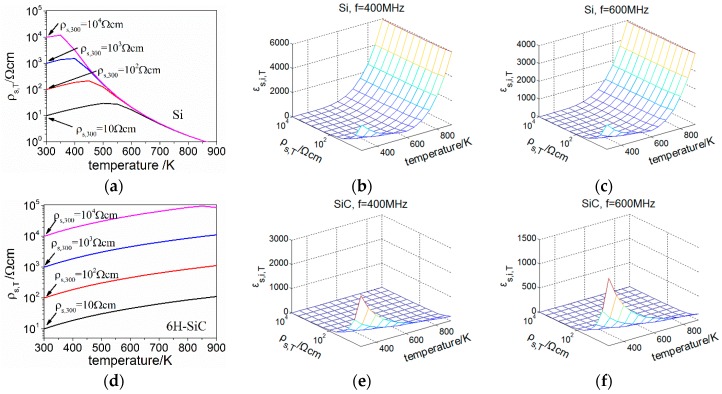
Calculated $\rho_{s,T}$ and $\varepsilon_{s,i,T}$ with different $\rho_{s,300}$ and temperatures (**a**) $\rho_{s,T}$ of Si; (**b**) $\varepsilon_{s,i,T}$ of Si at 400 MHz; (**c**) $\varepsilon_{s,i,T}$ of Si at 600 MHz; (**d**) $\rho_{s,T}$ of 6H-SiC; (**e**) $\varepsilon_{s,i,T}$ of 6H-SiC at 400 MHz; (**f**) $\varepsilon_{s,i,T}$ of 6H-SiC at 600 MHz.

Through Equations (25) and (27), the calculated $\rho_{s,T}$ and $\varepsilon_{s,i,T}$ with different $\rho_{s,300}$ and temperatures (300–900 K) are shown in Figure 5.

As shown in Figure 5a, $\rho_{s,T}$ of Si increases slightly and then decreases as the temperature rises, indicating a collaborative effect from the decreasing carrier mobility and increasing electron concentration. At temperatures higher than 600 K, the Si substrate is highly intrinsic, and $\rho_{s,T}$ with different $\rho_{s,300}$ become converged. Besides, both of the calculated $\varepsilon_{s,i,T}$ of Si at 400 MHz and 600 MHz are showing an increase on orders of magnitude at temperature higher than 600 K, mainly due to the decreasing $\rho_{s,T}$. As can be seen from Figure 5d, $\rho_{s,T}$ of 6H-SiC with different $\rho_{s,300}$ increases through the temperature range of calculation, and shows no convergence of the curves. As for $\varepsilon_{s,i,T}$ of 6H-SiC, it doesn’t manifest the increase at elevated temperatures observed in the case of Si. The different behaviors between Si and 6H-SiC are contributed to their different band gaps. 6H-SiC, with a wide band gap, shows less diversification of its electrical properties within the calculated temperature range.

Referring to Equations (1), (2), (8), (9) and (11)–(14), it can be seen that the geometric parameters are on the same order with $\rho_{s,T}$ or ε_s,c,T_, and considering that Si’s and 6H-SiC’s coefficients of thermal expansion range from around 2 ppm/C to 5 ppm/C in the temperature range of 300 to 900 K [28,29], which is much lower than the relative changes of $\rho_{s,T}$ or ε_s,c,T_, the effect from thermal expansion can be neglected.

**Figure 6:**
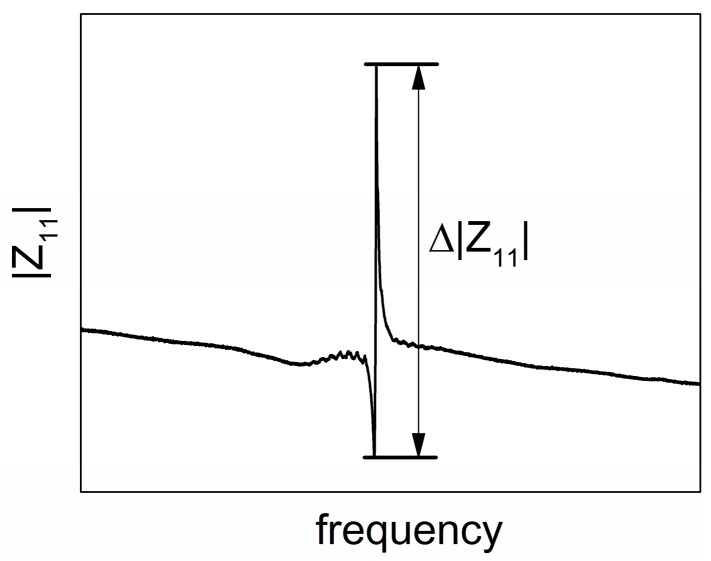
Typical $\left|Z_{11}\right|$ curve of SAWR in frequency domain.

With the calculated $\rho_{s,T}$ and $\varepsilon_{s,i,T}$, the temperature performance of SAWR can thus be derived through the modified equivalent circuit model introduced in this work. The geometric parameters and material properties used in the calculation are listed in Table A1 of Appendix A. And molybdenum (Mo) is chosen as the metal material for IDT and reflectors for its high melting point and close lattice constants with AlN. To evaluate the performance fluctuation of SAWR, the magnitude of the impedance ($\left|Z_{11}\right|$) is utilized. A typical frequency domain $\left|Z_{11}\right|$ curve of SAWR in frequency domain is shown in Figure 6, from which the serial and parallel resonant frequencies can be derived. In this work, $\Delta |Z_{11}|$, which is the difference between the $\left|Z_{11}\right|$ at both the serial and parallel resonant frequencies, is used to indicate the strength of the electrical tuning of the SAWR.

**Figure 7:**
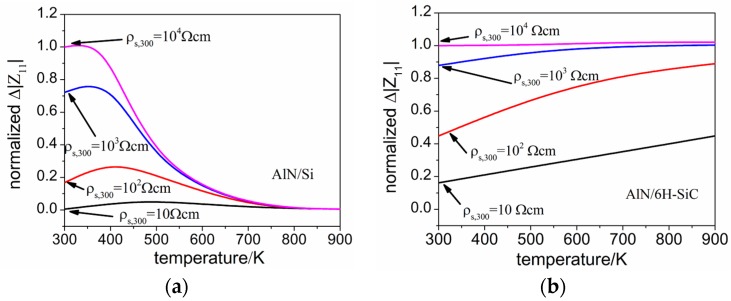
Temperature dependences of $\Delta |Z_{11}|$ for both AlN/Si and AlN/6H-SiC based SAWRs (**a**) AlN/Si based SAWR; (**b**) AlN/6H-SiC based SAWR.

SAWRs with configurations of AlN/Si and AlN/6H-SiC are modeled within a temperature range from 300 K to 900 K, the temperature dependence of $\Delta |Z_{11}|$ for each configuration is calculated and normalized by using the result when $\rho_{s,300}=10^{4}$ Ωcm and T = 300 K as a reference. As can be seen from Figure 7a,b, a decrease of $\Delta |Z_{11}|$ is shown in AlN/Si SAWR for every initial $\rho_{s,300}$, while in the case of AlN/6H-SiC, opposite responses are observed, indicating a more stable device performance could be achieved by using 6H-SiC as the substrate. The contrary performances between the two configurations can be contributed to the different electrical characteristics with temperature of Si and 6H-SiC, which has been discussed above.

### 2.5. Temperature Response of Center Frequency

**Figure 8:**
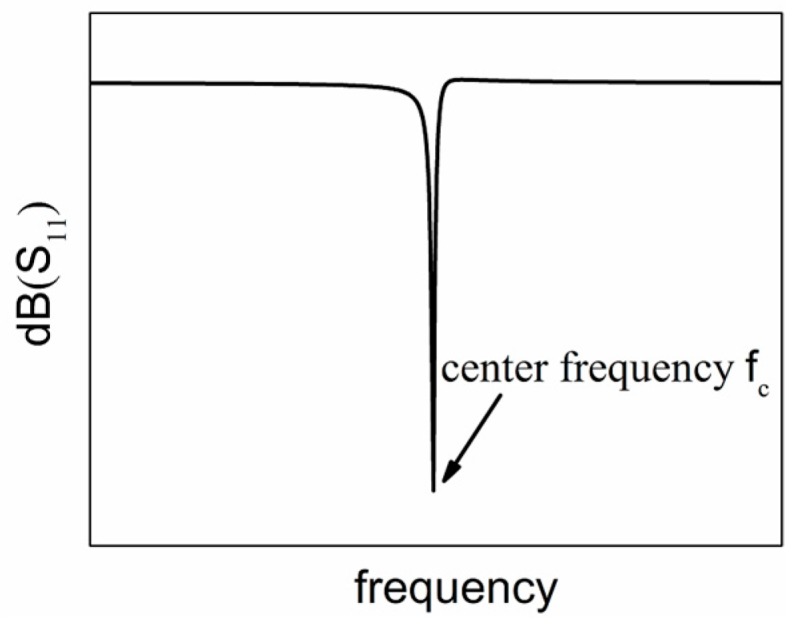
Typical S_11_ curve of SAWR in frequency domain.

Except for the electrical performance of SAWR discussed in previous section, the TCF is another important parameter when evaluating the temperature response to SAWR devices. In this work, the TCF of SAWR’s center frequency ($f_{c}$) is analyzed theoretically and experimentally. The center frequency can be derived through the frequency domain reflection coefficient (S_11_), corresponding to the frequency with the lowest S_11_ as shown in Figure 8.

For the theoretical analysis of $f_{c}$, it can be calculated by the SAW velocity ($v_{\mathrm{SAW}}$) and wavelength ($\lambda_{\mathrm{SAW}})$ as:

(29) $$f_{c}=v_{\mathrm{SAW}}/\lambda_{\mathrm{SAW}}$$

Considering $\lambda_{\mathrm{SAW}}$ equals the geometric period of IDT (λ), which along with $v_{\mathrm{SAW}}$, are all functions of temperature, therefore Equation (29) can further be expressed as:

(30) $$f_{c,T}=v_{\mathrm{SAW},T}/\lambda_{T}$$

By taking the center frequency at 300 K ($f_{c,300}$) as a reference, the TCF of SAR (${\mathrm{TCF}}_{\mathrm{SAW}}$) can be expressed as:

(31) $${\mathrm{TCF}}_{\mathrm{SAW}}=\frac{f_{c,T}-f_{c,300}}{f_{c,300}\Delta T}=\frac{1}{\Delta T}\frac{v_{\mathrm{SAW},T}-v_{\mathrm{SAW},300}}{v_{\mathrm{SAW},300}}-\frac{1}{\Delta T}\frac{\lambda_{T}-\lambda_{300}}{\lambda_{300}}$$

where $v_{\mathrm{SAW},300}$ and $\lambda_{300}$ are the SAW velocity and geometric period of IDT at 300 K, respectively.

The first term of Equation (31) represents the TCF of SAW velocity. For the hexagonal crystallized and cubic crystallized materials (Si, 6H-SiC, AlN) discussed in this work, the SAW velocity can be expressed by the mass density (ρ) and the elastic constants ($c_{\mathrm{ij}}$) as [30,31]:

(32) $$c_{33}c_{55}{\left({\rho v}_{\mathrm{SAW}}\right)}^{2}\left(c_{11}\;-{\rho v}_{\mathrm{SAW}}\right)=\left(c_{55}-{\rho v}_{\mathrm{SAW}}\right){{[c}_{33}{(c}_{11}\;-{\rho v}_{\mathrm{SAW}})-{c_{13}}^{2}]}^{2}$$

By taking temperature into consideration, $v_{\mathrm{SAW},T}$ can thus be expressed as:

(33) $$v_{\text{SAW,T}}=f(\rho_{T}{,c}_{\text{ij,T}})$$

where $\rho_{T}$ is the mass density under temperature T and can be given by:

(34) $$\rho_{T}=\rho_{300}\left(1-\Delta T\underset{m}{\sum }\bar{a_{T,m}}\right)$$

$\rho_{300}$ is the mass density at 300 K, $\bar{a_{T,m}}$ is the effective thermal expansion coefficient of the material along m-axis (m = x, y, z), representing the average expansion rate through 300 K to (300 + ΔT)K, and can be calculated as:

(35) $$\bar{a_{T,m}}=\frac{1}{\Delta T}\int_{300}^{300+\Delta T}a_{T,m}\left(T\right)\mathrm{dT}$$

$c_{\text{ij,T}}$ in Equation (33) represents the elastic constants under temperature T, and can be calculated by:

(36) $$c_{\text{ij,T}}=c_{\mathrm{ij},300}\left(1+\Delta T\frac{{\mathrm{dc}}_{\mathrm{ij}}}{c_{\mathrm{ij}}\mathrm{dT}}\right)$$

For the second term of Equation (31), noticing that in the coordinate system defined in Figure 1, SAW propagates along the x-axis, hence $\lambda_{T}$ can be expressed as:

(37) $$\lambda_{T}=\lambda_{300}\left(1+\bar{a_{T,x}}\Delta T\right)$$

By taking Equations (37) into (31), Equation (31) can further be expressed as:

(38) $${\mathrm{TCF}}_{\mathrm{SAW}}=\frac{1}{\Delta T}\frac{v_{\mathrm{SAW},T}-v_{\mathrm{SAW},300}}{v_{\mathrm{SAW},300}}-\bar{a_{T,x}}$$

The ${\mathrm{TCF}}_{\mathrm{SAW}}$ of AlN, Si, 6H-SiC can thus be calculated and shown in Table 3, in addition to the parameters listed in Table A1 of Appendix A, all other parameters used in calculation have been listed in Table A2 of Appendix A [28,29,32,33,34,35,36].

**Figure 9:**
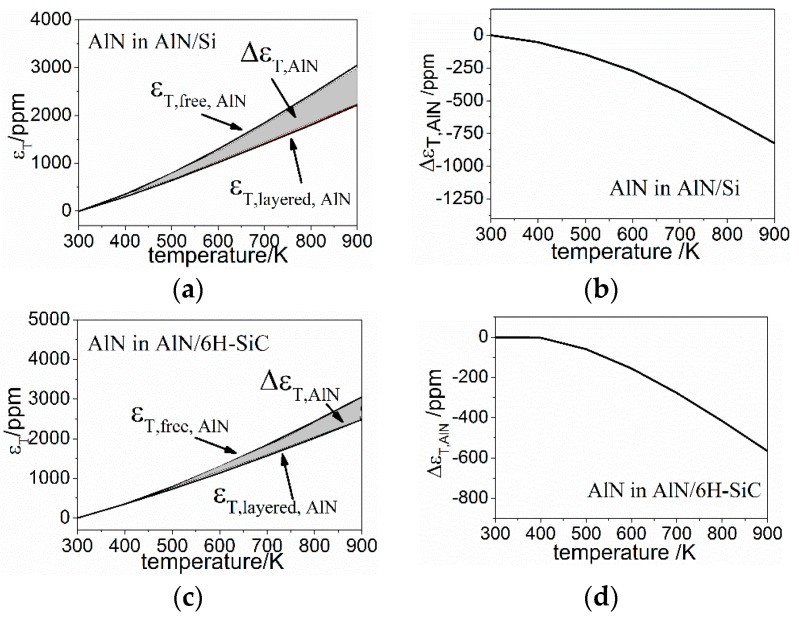
Temperature dependences of $\Delta \varepsilon_{T}$ in AlN layer for both AlN/Si and AlN/6H-SiC based SAWRs (**a**,**b**) AlN in AlN/Si based SAWR; (**c**,**d**) AlN in AlN/6H-SiC based SAWR.

It is known that the energy of SAW is constrained within several wavelengths beneath the surface of IDT plane [18], hence with a wavelength larger than the thickness of AlN in this work, the actual ${\mathrm{TCF}}_{\mathrm{SAW}}$ of AlN/Si and AlN/6H-SiC based SAWR will be decided by both the piezoelectric film (AlN) and the substrate (Si or 6H-SiC). Besides, the mismatch of thermal expansions between AlN and the substrate in the x-y plane will also introduce thermal mismatch strains, namely $\Delta \varepsilon_{T}$, which can be calculated by the thermal expansion of material in free and layered conditions, as:

(39) $$\Delta \varepsilon_{T}=\varepsilon_{T,\mathrm{free}}-\varepsilon_{T,\mathrm{layered}}$$

where $\varepsilon_{T,\mathrm{free}}$ is the ideal thermal expansion of free material, and $\varepsilon_{T,\mathrm{layered}}$ is the thermal expansion of material in the layered structure. Consideringthe much larger thickness of the substrate than that of AlN, the thermal expansions of the AlN/Si and AlN/6H-SiC based SAWRs are decided by the substrates, hence the thermal mismatch strains of the substrates can be neglected, and the strain of AlN is not sensitive to the film thickness, both of which has been confirmed by finite element modeling results. For each of the AlN layers in the calculated AlN/Si and AlN/6H-SiC based SAWRs, with the premise that it is isotropic in the x-y plane, the $\Delta \varepsilon_{T}$ of each configuration is calculated and shown in Figure 9.

With the given strain field $\varepsilon =\left[\Delta \varepsilon_{T},\Delta \varepsilon_{T},0\right]$, the relative change of $f_{c}$ can thus be calculated by:

(40) $$\frac{\Delta f_{c}}{f_{c}}=\frac{f_{c,\varepsilon }-f_{c,0\varepsilon }}{f_{c,0\varepsilon }}=\frac{v_{\mathrm{SAW},\varepsilon }\;-v_{\mathrm{SAW},0\varepsilon }}{v_{\mathrm{SAW},0\varepsilon }}\;-\;\frac{\lambda_{\varepsilon }\;-\lambda_{0\varepsilon }}{\lambda_{0\varepsilon }}$$

Therefore the TCF introduced by the thermal strain mismatch can be expressed as:

(41) $${\mathrm{TCF}}_{\varepsilon }=\frac{f_{c,\varepsilon }-f_{c,0\varepsilon }}{\Delta {\mathrm{Tf}}_{c,0\varepsilon }}=\frac{v_{\mathrm{SAW},\varepsilon }\;-v_{\mathrm{SAW},0\varepsilon }}{\Delta {\mathrm{Tv}}_{\mathrm{SAW},0\varepsilon }}\;-\;\frac{\lambda_{\varepsilon }\;-\lambda_{0\varepsilon }}{\Delta {T\lambda }_{0\varepsilon }}$$

where $\lambda_{\varepsilon }$ and $\lambda_{0\varepsilon }$ are the geometric period of IDT with strain ε and without ε, and has the relationship:

(42) $$\lambda_{\varepsilon }{=\lambda }_{0\varepsilon }(1+\Delta \varepsilon_{T})$$

Similarly, $v_{\mathrm{SAW},\varepsilon }$ and $v_{\mathrm{SAW},0\varepsilon }$ are, respectively, the SAW velocity under the appearance and absence of strain ε. As shown in Equation (32), $v_{\mathrm{SAW}}$ can be given by the elastic constant and the mass density, which are both functions of strain field ε, therefore $v_{\mathrm{SAW},\varepsilon }$ can be given as:

(43) $$v_{\mathrm{SAW},\varepsilon }=f(\rho_{\varepsilon }{,c}_{\mathrm{ij},\varepsilon })$$

where $\rho_{\varepsilon }$ is the mass density under strain field and can be given by:

(44) $$\rho_{\varepsilon }=\rho_{0\varepsilon }(1-2\Delta \varepsilon_{T})$$

where $c_{\mathrm{ij},\varepsilon }$ represents the elastic constants of AlN in strain field, and can be calculated by the third order elastic constants $c_{\mathrm{ijk}}$ as given by references [36,37,38] and written as Equation (45) for simplicity. The third order elastic constants used for calculation has been in Table A2 of Appendix A.

(45) $$c_{\mathrm{ij}}\left(\varepsilon \right)=f(c_{\mathrm{ij}}{,c}_{\mathrm{ijk}},\varepsilon )$$

The calculated ${\mathrm{TCF}}_{\varepsilon }$ of AlN in AlN/Si and AlN/6H-SiC configurations are −0.03 ppm/K and −0.02 ppm/K.

As can be seen, the ${\mathrm{TCF}}_{\varepsilon }$ of AlN in both of the calculated configurations are orders of magnitude lower than the intrinsic TCF_SAW_ of AlN listed in Table 3, therefore the ${\mathrm{TCF}}_{\varepsilon }$ has little influence on the total TCF of the AlN layer in either SAWR configuration.

## 3. Experimental Section

The fabricated AlN/Si and AlN/6H-SiC SAWRs in this work has the same layout design, as has been given by Table A1 of Appendix A.

For the AlN/Si configuration, a 400 μm thick, 4″ n-type (100) silicon wafer is prepared as substrate, and cleaned with buffer hydrofluoric acid (BHF), acetone, isopropanol (IPA) and deionized water. The resistivity of the substrate is around 4.5 × 10^3^ Ωcm (4-Probe method, NPS RESISTEST VIII). Polycrystalline AlN layer with a thickness of 3 μm is deposited on the Si substrate via RF sputtering, followed by 100 nm thick of Mo deposited with the same method. The Mo layer is patterned into the designed layout by reactive ion etching (RIE). A gold (Au) layer of 100 nm in thickness is then evaporated and patterned as electrodes for signal connection.

As for the AlN/6H-SiC configuration, a 350 μm thick, 4″ n-type (0002) 6H-SiC wafer is chosen as the substrate, with a nominal resistivity higher than 1 × 10^5^ Ωcm. The same micro fabrication process is utilized for fabricating the SAWR as in the case of AlN/Si configuration. It should be noted that besides the thickness of the substrate, the thickness of AlN film in AlN/6H-SiC configuration, being 1.5 μm, is also different from that of AlN/Si configuration due to the fabrication capability. But since this work mainly focuses on the relative fluctuation of each configuration under different temperatures, these inconsistencies will have little effect on the results and conclusions.

An abbreviated fabrication process has been illustrated in previously reported work [38]. The AlN films in both configurations are characterized by X-ray diffraction (XRD) spectrum, and the Mo/AlN/substrate layered stacks are characterized by scanning electrons microscope (SEM).

After the micro-fabrication process, SAWRs are diced from wafers and adhered to connection board by high temperature adhesion agent. High temperature silver paste (SX-9302) is used to electrically connect the SAWR devices and connections boards.

**Figure 10:**
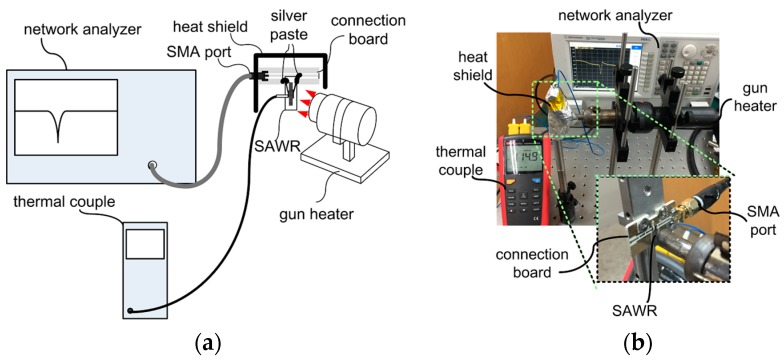
Apparatus used for temperature response testing (**a**) Designed diagram; (**b**) Photo.

The high temperature range responses of the SAWRs are then tested from 300 to 725 K with the apparatus shown in Figure 10. The SAWRs are heated by a gun heater (ATTEN instrument), with the temperature monitored by thermocouples (Uni-Trend Technology, UT322). The frequency domain response of the SAWRs is monitored and recorded by network analyzer (Agilent Technology, N5230C) simultaneously. Temperature responses of both electrical performance and center frequency are evaluated and compared with the modeling and calculated results.

## 4. Results and Discussion

**Figure 11:**
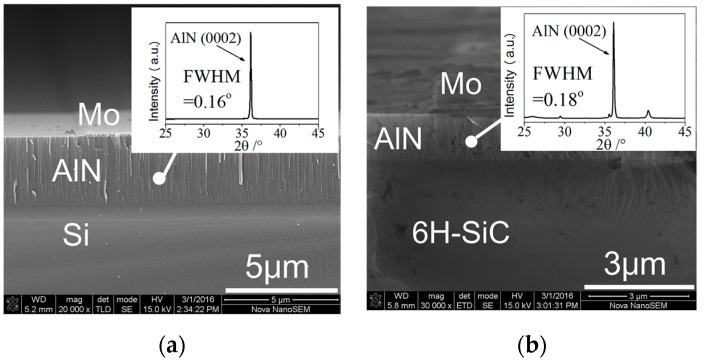
Characterization of the layered SAWR device (**a**) XRD and SEM characterization of AlN/Si configuration; (**b**) XRD and SEM characterization of AlN/6H-SiC configuration.

The XRD and SEM characterizations of the as-deposited AlN on both Si and 6H-SiC are shown in Figure 11. The full width at half maximum (FWHM) of the (0002)-AlN can be derived from the XRD spectrum and are 0.16° and 0.18° for the AlN/Si and AlN/6H-SiC configuration, respectively, indicating highly c-axis oriented (0002) AlN has been grown on each substrate.

**Figure 12:**
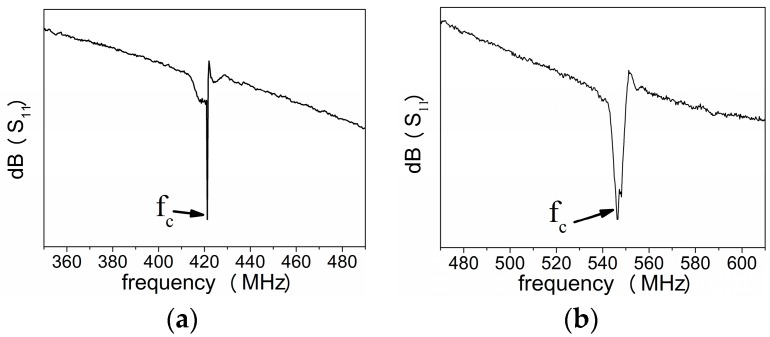
Typical reflection coefficient of the AlN/Si SAWR in this work (**a**) AlN/Si based SAWR; (**b**) AlN/6H-SiC based SAWR.

The center frequency of the fabricated AlN/Si SAWR is 422 MHz, while that of AlN/6H-SiC is 546 MHz. The typical reflection coefficient S_11_ of both configurations have been shown in Figure 12.

**Figure 13:**
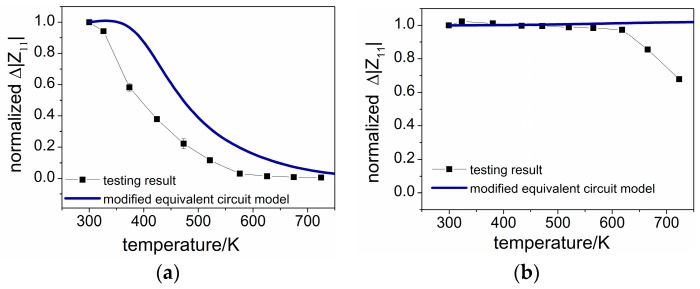
Electrical performances of SAWRs under different temperatures (**a**) AlN/Si based SAWR; (**b**) AlN/6H-SiC based SAWR.

The electrical performances of SAWRs under different temperatures are evaluated by analyzing the frequency domain response of each configuration. Similar to the method mentioned in the modeling part, $\Delta |Z_{11}|$ of each temperature point is used for the comparison, and the values of $\Delta |Z_{11}|$ are normalized by using the room temperature (300 K) data for comparison. The tested results are shown in Figure 13a,b.

As can be seen, the $\Delta |Z_{11}|$ of AlN/Si configuration decreases fast with an increasing temperature, while that for AlN/6H-SiC configuration degrades less than 5% within 625 K, which are in accordance with the predicted performances given by the modified equivalent circuit model.

The differences between the modeling and experimental results may come from the serial parasitic parameters such as the resistors and inductors introduced by the IDT grids and the conducting lines, which are not considered in the modeling here but also vary with temperature. Another possible contributing factor to the differences is the lattice vibration [39], leading to stronger acoustic scattering and phonon loss at elevated temperatures, which may lead to a deterioration stronger than the modeling results and play a dominating role at higher temperatures, as shown in the AlN/6H-SiC configuration at temperatures higher than 650 K.

After all, based on the modeling and experimental results shown in this work, it can be inferred that the semiconductor property of the substrate, mostly the band gap, is playing a key role in the electrical performance of the AlN-film based SAWR, and substrates with a higher band-gap will lead to a more stable high temperature performance.

**Figure 14:**
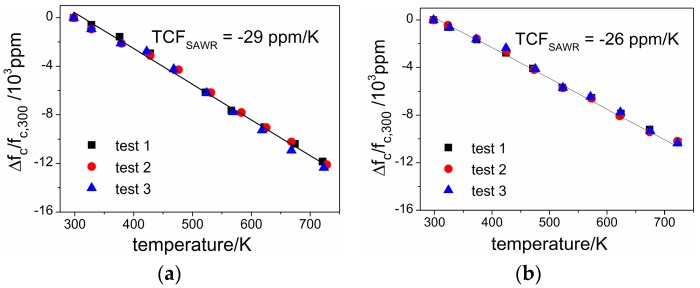
Electrical performances of SAWRs under different temperature (**a**) AlN/Si based SAWR; (**b**) AlN/6H-SiC based SAWR.

The temperature responses of center frequency are also tested for both configurations and linear approximation is used for deriving the first order TCF_SAWR_, as shown in Figure 14. The first order TCF_SAWR_ of −29 ppm/K is obtained for the AlN/Si configuration, and −26 ppm/K for the AlN/SiC one. As has been shown in Section 2.5, the frequency shift due to thermal mismatch is negligible compared to the intrinsic TCF_SAW_ of the materials discussed in this work, therefore the differences between the calculated TCF_SAWR_ (−26~−28 ppm/K for the AlN/Si configuration and −25~−28 ppm/K for the AlN/6H-SiC configuration) can be contributed to the inconformity of the actual parameter and those used in calculation, especially the temperature coefficient factors of elastic constants ($\frac{1}{\Delta T}\frac{\Delta c_{\mathrm{ij}}}{c_{\mathrm{ij}}}$), which are sensitive to testing methods and have existing inconsistencies among different research reports.

Considering the relative mismatch of the calculated TCF_SAWR_ to the experimental one is less than 15% in each configuration, the method induced in this work is an effective way for estimating the TCF of AlN-film based SAWRs, which would be important when designing SAWR-based sensors working in a wide temperature range and if temperature compensation being needed.

## 5. Conclusions

The temperature responses of aluminum nitride (AlN) film based surface acoustic wave resonators (SAWR) are modeled and tested. A modified equivalent circuit model is introduced to model the electrical performance in a varying temperature environment. By taking the parasitic parameters from the substrate into the modeling, the electrical performances of the AlN-film based SAWRs can be predicted, and are further compared with high temperature experiments from 300 K to 725 K, leading to a conclusion that SAWR based on substrates with wider band gaps will lead to a more stable high temperature behavior.

Temperature responses of SAWR’s center frequency are also calculated and tested, with experimental TCF_SAWR_ of center frequencies being −29 ppm/K and −26 ppm/K for the AlN/Si and AlN/6H-SiC based SAWRs. Considering the relative mismatch is less than 15% between calculated TCF_SAWR_ and the experimental ones, the calculation method introduced in this work is an effective way for estimating the TCF of AlN-film based SAWRs, which would be important when designing SAWR-based sensors working in a wide temperature range as well as temperature compensation.

## Figures and Tables

**Table 1:** Material parameters used in calculating $\rho_{s,T}$ [25,27].

Parameters	Value	Unit	Parameters	Value	Unit
$\mu_{300,\mathrm{Si}}$	1350	cm/Vs	$N_{C0,\mathrm{Si}}$	5.41 × 10^15^	cm^−3^K^−3/2^
$\mu_{300,\mathrm{SiC}}$	400	cm/Vs	$N_{V0,\mathrm{Si}}$	2.19 × 10^15^	cm^−3^K^−3/2^
$\rho_{s,\mathrm{Si},300}$	10, 10^2^, 10^3^, 10^4^	Ωcm	$N_{C0,\mathrm{SiC}}$	17.3 × 10^15^	cm^−3^K^−3/2^
$\rho_{s,\mathrm{SiC},300}$	10, 10^2^, 10^3^, 10^4^	Ωcm	$N_{V0,\mathrm{SiC}}$	4.80 × 10^15^	cm^−3^K^−3/2^
$E_{g,\mathrm{Si}}$	1.12	eV	e	1.6 × 10^−19^	C
$E_{g,\mathrm{SiC}}$	3.0	eV	k	1.3806 × 10^−23^	J/K

**Table 2:** Calculated $N_{d}$ with different $\rho_{s,300}$ (300 K).

$\rho_{s,Si,300}$/Ωcm	$N_{d,Si}$/cm^−3^	$\rho_{s,SiC,300}$/Ωcm	$N_{d,SiC}$/cm^−3^
10	4.63 × 10^14^	10	1.56 × 10^15^
10^2^	4.63 × 10^13^	10^2^	1.56 × 10^14^
10^3^	4.63 × 10^12^	10^3^	1.56 × 10^13^
10^4^	4.63 × 10^11^	10^4^	1.56 × 10^12^

**Table 3:** Calculated ${\mathrm{TCF}}_{\mathrm{SAW}}$ of AlN, Si, and 6H-SiC.

Material	${\mathrm{TCF}}_{SAW}/ppmK^{-1}$
AlN	−28
Si	−26
6H-SiC	−25

## Abbreviations

The following abbreviations are used in this manuscript:

SAWR	Surface acoustic wave resonator
TCF	Temperature coefficient factor
IDT	Inter-digital transducer
FWHM	Full width at half maximum

## Appendix A

The geometric and material parameters used for modeling and calculation in this work are listed in Table A1.

**Table A1:** Material parameters used for modeling and calculation (C_ij_ represents the elastic constants of material, i,j = 1~6).

Parameters	Value/Unit	Parameters	Value/Unit
c_11,AlN_	408.03/GPa	c_44,SiC_	137/GPa
c_12,AlN_	100.18/GPa	c_66,SiC_	153/GPa
c_13,AlN_	83.40/GPa	$\rho_{300,\mathrm{SiC}}$	3216/kg·m^−3^
c_33,AlN_	384.30/GPa	$\varepsilon_{\mathrm{SiC},R}$	9.7
c_44,AlN_	100.08/GPa	t_s,Si_	400/μm
c_66,AlN_	153.93/GPa	t_p,AlN-Si_	3/μm
$\rho_{300,\mathrm{AlN}}$	3260/kg·m^−3^	t_s,SiC_	400/μm
$\varepsilon_{\mathrm{AlN},R}$	9	t_p,AlN-SiC_	3/μm
c_11,Si_	165.63/GPa	t_m_	100/nm
c_12,Si_	63.87/GPa	N	50
c_44,Si_	79.55/GPa	λ	12/μm
$\rho_{300,\mathrm{Si}}$	2329/kg·m^−3^	a	3/μm
c_11,Mo_	440.77/GPa	w	840/μm
c_12,Mo_	172.43/GPa	N_R_	600
c_44,Mo_	121.65/GPa	l_c1_	600/μm
$\rho_{\mathrm{Mo}}$	10,200/kg·m^−3^	l_c2_	5000/μm
$\varepsilon_{\mathrm{Si},R}$	11.7	w_c_	200/μm
c_11,SiC_	493/GPa	l_t_	7.5/μm
c_12,SiC_	187/GPa	w_cr_	480/μm
c_13,SiC_	91/GPa	w_i_	40/μm
c_33,SiC_	553/GPa	K^2^	0.2%

**Table A2:** Material parameters used for modeling and calculation (complementary parameters) [28,29,32,33,34,35,37].

Parameters	Value/Unit	Parameters	Value/Unit
$a_{T,x,\mathrm{AlN}}$	ref. [32]	$\frac{1}{\Delta T}\frac{\Delta c_{13,\mathrm{Si}}}{c_{13,\mathrm{Si}}}$	ref. [34]
$a_{T,y,\mathrm{AlN}}$	ref. [32]	$\frac{1}{\Delta T}\frac{\Delta c_{11,\mathrm{SiC}}}{c_{11,\mathrm{SiC}}}$	ref. [35]
$a_{T,z,\mathrm{AlN}}$	ref. [32]	$\frac{1}{\Delta T}\frac{\Delta c_{33,\mathrm{SiC}}}{c_{33,\mathrm{SiC}}}$	ref. [35]
$a_{T,x,\mathrm{Si}}$	ref. [28]	$\frac{1}{\Delta T}\frac{\Delta c_{44,\mathrm{SiC}}}{c_{44,\mathrm{SiC}}}$	ref. [35]
$a_{T,y,\mathrm{Si}}$	ref. [28]	$\frac{1}{\Delta T}\frac{\Delta c_{13,\mathrm{SiC}}}{c_{13,\mathrm{SiC}}}$	ref. [35]
$a_{T,z,\mathrm{Si}}$	ref. [28]	c_111,AlN_	−3072.30/GPa
$a_{T,x,\mathrm{SiC}}$	ref. [29]	c_112,AlN_	−514.07/GPa
$a_{T,y,\mathrm{SiC}}$	ref. [29]	c_113,AlN_	−75.06/GPa
$a_{T,z,\mathrm{SiC}}$	ref. [29]	c_123,AlN_	−155.12/GPa
$\frac{1}{\Delta T}\frac{\Delta c_{11,\mathrm{AlN}}}{c_{11,\mathrm{AlN}}}$	−37/ppmK^−1^	c_133,AlN_	−614.88/GPa
$\frac{1}{\Delta T}\frac{\Delta c_{33,\mathrm{AlN}}}{c_{33,\mathrm{AlN}}}$	−65/ppmK^−1^	c_344,AlN_	−576.45/GPa
$\frac{1}{\Delta T}\frac{\Delta c_{44,\mathrm{AlN}}}{c_{44,\mathrm{AlN}}}$	−50/ppmK^−1^	c_144,AlN_	−150.12/GPa
$\frac{1}{\Delta T}\frac{\Delta c_{55,\mathrm{AlN}}}{c_{55,\mathrm{AlN}}}$	−50/ppmK^−1^	c_155,AlN_	−100.08/GPa
$\frac{1}{\Delta T}\frac{\Delta c_{13,\mathrm{AlN}}}{c_{13,\mathrm{AlN}}}$	−1.8/ppmK^−1^	c_222,AlN_	−2413.0/GPa
$\frac{1}{\Delta T}\frac{\Delta c_{11,\mathrm{Si}}}{c_{11,\mathrm{Si}}}$	ref. [34]	c_333,AlN_	−2213.6/GPa
$\frac{1}{\Delta T}\frac{\Delta c_{44,\mathrm{Si}}}{c_{44,\mathrm{Si}}}$	ref. [34]		
